# A critique of current approaches to privacy in machine learning

**DOI:** 10.1007/s10676-025-09843-4

**Published:** 2025-06-20

**Authors:** Florian van Daalen, Marine Jacquemin, Johan van Soest, Nina Stahl, David Townend, Andre Dekker, Inigo Bermejo

**Affiliations:** 1 Radiation Oncology (MAASTRO) GROW School for Oncology and Reproduction, Maastricht University Medical Centre, Maastricht, Netherlands; 2 Department of Health Promotion, Care and Public Health Research Institute (CAPHRI), Maastricht University, Maastricht, Netherlands; 3 City Law School, University of London, London, United Kingdom; 4 Data Science Institute, Hasselt University, Hasselt, Belgium; 5 Brightlands Institute for Smart Society (BISS), Faculty of Science and Engineering, Maastricht University, Maastricht, Netherlands; 6 Department of Health, Ethics and Society (HES), Faculty of Health, University Maastricht, Maastricht, Netherlands

**Keywords:** Privacy, Ethics, Machine learning, Privacy preserving

## Abstract

Access to large datasets, the rise of the Internet of Things (IoT) and the ease of collecting personal data, have led to significant breakthroughs in machine learning. However, they have also raised new concerns about privacy data protection. Controversies like the Facebook-Cambridge Analytica scandal highlight unethical practices in today’s digital landscape. Historical privacy incidents have led to the development of technical and legal solutions to protect data subjects’ right to privacy. However, within machine learning, these problems have largely been approached from a mathematical point of view, ignoring the larger context in which privacy is relevant. This technical approach has benefited data-controllers and failed to protect individuals adequately. Moreover, it has aligned with Big Tech organizations’ interests and allowed them to further push the discussion in a direction that is favorable to their interests. This paper reflects on current privacy approaches in machine learning and explores how various big organizations guide the public discourse, and how this harms data subjects. It also critiques the current data protection regulations, as they allow superficial compliance without addressing deeper ethical issues. Finally, it argues that redefining privacy to focus on harm to data subjects rather than on data breaches would benefit data subjects as well as society at large.

## Background

The promise to deliver innovation in fields as diverse as healthcare, transportation and education has made it difficult to ignore the appeal of collecting and processing vast amounts of personal data. Access to large datasets, the rise of the Internet of Things (IoT), and the ease of collecting personal data, have led to significant breakthroughs in machine learning (Kairouz et al. 2021; Xu et al. 2021; Hagen 2021; Truong et al. 2021). However, they have also raised concerns about privacy data protection (Bak et al. 2024). Awareness of privacy issues in the era of Big Data is growing, fuelled by recent controversies such as the Facebook-Cambridge Analytica scandal (Hinds et al. 2020; ur Rehman 2019), Clear View AI (Ahmed 2023), and reports from privacy watchdogs like the Mozilla Foundation (Foundation 2023), which highlighted the unethical practices that have become commonplace in today’s digital landscape. Personal data is extremely valuable (Palmer 2005) and often harvested without the knowledge or consent of individuals, leading to potentially negative consequences, not only for them, but also for society as a whole.

Partially in response to these concerns, European lawmakers adopted the General Data Protection Regulation (GDPR) in 2016 (GDPR 2016). In the US, the State of California soon followed suit, implementing the Californian Consumer Privacy Act (CCPA) in California Code Civil Code (2023), soon amended by the California Privacy Right Act (CPRA). These two acts however only apply to Californians, and there is no federal-level data protection regulation in the United States outside of the Health Insurance Portability and Accountability Act (HIPAA), which only applies to health data. Both the GDPR and CCPA provide enforceable rights to data subjects and clearly define the notion of lawful data processing, with real repercussions in case of non-compliance (EDPB 2023; GDPR Enforcement Tracker 2024; Register 2024; GDPR 2016; Euronews 2022; Cromack 2021; Euronews 2022).^1^ This formed the perfect context to encourage the further development of so-called “privacy-preserving” data analysis solutions enabling machine learning models to be trained without compromising privacy and therefore avoiding data protection related fines. Various metrics, such as k-anonymity Sweeney (2002), sensitivity and $\epsilon$-differential privacy Dwork and Roth (2015) and Dwork et al. (2006), have been established, advertised as ways to measure such privacy-preservation in an objective and generalizable manner.

This paper aims to critically reflect on the current approaches to privacy in machine learning. First, we will briefly introduce the concept of privacy as understood within social science and law. We will argue that privacy has increasingly been approached as a mathematical concept, explaining how this technical approach, while beneficial for data-controllers, fails to protect the interests of data subjects. Next, we will consider the role of Big Tech in defining what should or should not be considered private, and how their influence significantly impacts the social understanding of privacy. Finally, we will discuss how the current situation might be improved to benefit individuals and society as large, by arguing for a shift from privacy-preserving machine learning towards an approach focused on risk assessment and harm mitigation.

## What even is privacy?

To meaningfully assess the question of privacy preservation, we must first understand the concept of privacy itself. This concept is complex, multi-faceted and context-dependent, having been explored extensively among disciplines such as social science, law, and computer science. This section will focus on the first two, offering the knowledge basis necessary to understand how they differ from the understanding of privacy common within data science and machine learning. One of the first known definitions of privacy in law was given in 1890 by Warren and Brandeis Warren and Brandeis (1890), presenting it as “the right to be left alone”, as a response to the increasingly intrusive behavior of the newspapers and paparazzi of the time. Their work catalyzed a broader conversation around individual control over access to one’s personal life. Since then, layered and evolving understandings of privacy have been developed within social science. Different disciplinary understandings of privacy’ can be summarized as frameworks developed to present the key nuances of privacy as a concept, and how it was shaped by our contemporary society. Indeed, privacy being a flexible and evolving concept Tavani (2008), it evades a clearly set definition.

That privacy is conceptually contested between and within disciplines needs further explanation. There is little agreement about the limits of privacy.^2^ This can be explained by the dynamic nature of privacy; by its nature it is personal, defines the world of the individual claiming the right, and is defined to a very large extent by the person claiming the right. We agree the first line, “we have a human right to privacy”, but there is very little agreement about the granularity of privacy thereafter—there is no agreed second line, “this is your privacy”.^3^ When one examines the case law on privacy of, for example, the European Court of Justice, privacy disputes are resolved, but this is arguably a dispute resolution between two parties where privacy is identified when it is seen, rather than creating a canon of law that prescribes the granular detail of privacy as a normative template to use moving forward. European Court of Human Rights (2022) Fundamentally, is “privacy”—a right to a private life—a right that can or should be defined, or constrained? Privacy is about, Laurie argues, one’s space to be who one wishes to be, within broad (and not narrow) societal constraints.^4^ Laurie argues that privacy is the space where we negotiate our relationship with others in society. Townend has argued that the way of negotiating that space should be a claim, by the person seeking to challenge an individual’s privacy, to a necessity so to do in the public interest. He argues that one’s privacy can be ‘breached’ (as privacy in the international instruments is not an absolute right) where doing so is in a supervening public interest. This is not necessarily simply a Utilitarian approach. It can be argued as a question of reasonableness in Rawls, or an operation of the Categorical Imperative in making a claim to privacy without instrumentalising others.^5^

It is necessary to apply this conceptual perspective to more practical issues. One widely accepted perspective, closely tied to Warren & Brandeis’ initial definition, is the idea of privacy as a form of control (Moor 2006; Moore 2008; Macnish 2018), specifically over access to one’s body, communications, decisions, and personal data.^6^ Furthermore, one’s understanding of what is or should be private may differ based on context. For example, sharing personal health information with a doctor would not be considered an attack on privacy, whereas that same information being accessed by financial institutions would. This idea is the central thesis of Nissenbaum Nissenbaum (2009) and Roessler and DeCew (2023), who argues, through a concept known as contextual integrity, that our privacy expectations are shaped by the norms governing different social contexts, such as the doctor-patient relationship in our example. Privacy has also been closely linked to autonomy and freedom (Dean 2003; Bundesverfassungsgericht 1983; Solove 2002; Peloquin et al. 2020; Cohen 2012), with authors suggesting that attacks on privacy undermine individuals’ capacity to think and act independently. This concern is particularly relevant in the digital era, when information is increasingly being concentrated in only a few powerful data-driven institutions. Individuals being consistently monitored, profiled, or influenced based on personal information about them may self-censor or alter their behavior. Privacy can thus also be understood as a matter of power. Control over personal data gives power over not only these individuals, but also entire populations. This dynamic stands at the core of what Zuboff describes as “surveillance capitalism” Zuboff (2023), where personal information is commodified and used to predict ad manipulate behavior at a large scale. It highlights the importance of privacy as a way to maintain power, given that erosion of that right results in individual and systemic harm.

Social science and legal understanding of privacy have historically mutually shaped one another Roessler and DeCew (2023). European law explicitly recognizes privacy as a fundamental right, enshrining it in article 8 of the European Convention on Human Rights, which states “everyone has the right to respect for his or her private and family life, home and communications”. However, a precise definition of what constitutes such “privacy” from a legal perspective is lacking. In contrast, the closely entangled concept of “personal data” is clearly defined as data that can be linked back to an individual. As such, it forms the basis of European data protection regulation. This framework is often understood as a means to protect or safeguard privacy, despite data protection and privacy being often conflated but existing in the European Union as distinct fundamental rights (Fuster and Gellert 2012; Fuster 2014; European Union 2007). Indeed, it is entirely possible to abide by data protection regulation without respecting one’s right to privacy (De Hert and Gutwirth 2006; Nair and Tyagi 2021), one such scenarios include claiming a legitimate interest for data processing [art. 6 (1)(f) of the GDPR] and following simple procedural rules such as informing the data subject that their data will be monitored or collected. In well-argued cases of legitimate interest, obtaining consent is not required Data Protection Working Party (2014). In such instances, formal compliance can obscure significant breaches of privacy Cohen (2012). Understanding privacy through a strict data protection lens can therefore lead to a narrow approach that neglects the broader ethical considerations mentioned earlier in this section, such as control, autonomy and asymmetrical concentration of power.

## Privacy as a mathematical concept

One final understanding of “privacy” is its interpretation within the realm of machine learning and data science. Repercussions for non-compliance with the legal requirements set forth in the GDPR—such as ensuring the lawfulness of data processing, purpose limitation, data minimization and transparency obligations—can be severe. As a result, the regulation has sometimes had the unintended consequence of hindering data-sharing across institutions and EU member states, even for research purposes (Peloquin et al. 2020). Yet the appeal of conducting research based off large amounts of data processing has not diminished. As a response, technological solutions have been developed to reduce privacy leaks and thus data processing becomes more compliant with existing legislation. A few of these solutions include Multiparty Computation (MPC) (Yao 1982), Federated Learning (FL) (Dwork and Roth 2015), homomorphic encryption (Parmar et al. 2014), and synthetic data (van Daalen et al. 2024; Gregor et al. 2015; Denton et al. 2015; Eigenschink et al. 2023) to replace real data when training models. Finally, attempts to measure privacy in a concrete mathematical manner have been developed. In this context, privacy is approached mainly using the following three nonconflicting methods: (a) by setting and utilizing privacy thresholds, (b) by focusing on limiting data breaches, which we will discuss in “The misplaced focus on preventing data leaks and its consequences” section, and (c) through the use of so-called “privacy-preserving” technologies.

### Setting and utilizing privacy thresholds

Whether it is hypothesis testing with *p*-values (Amrhein et al. 2019), creating a sufficient level of privacy with a privacy budget using schemes like k-anonymity or $\epsilon$-differential privacy, or deciding if a model’s predictions are accurate enough, statistical measures use specific thresholds as cut-off points to determine if the scenario passes a test. However, these thresholds are often set based on historic precedent rather than any truly objective reasoning. While these thresholds can be informative to a certain extent, the focus on these historic precedents causes researchers to be mostly concerned with simply passing this threshold, which has resulted in several important problems.

First, researchers are often not aware of how and why these thresholds were set (Mitchell et al. 2010). This is especially true for researchers who are not statisticians themselves. For example, most researchers working with quantitative data know about *p*-values, but probably would not be able to explain why the common threshold used to indicate statistical significance was set to 0.05. Yet, they will still accept or dismiss research based on this threshold. Second, this can also tempt said researchers to tweak their experiments in various ways to pass this test, which might mislead research findings (Head et al. 2015; Bouter et al. 2016; John et al. 2012). In the context of privacy this may mean that a researcher may mindlessly accept a privacy solution because a statistical test shows that with $p<0.05$ no data is leaked. Lastly, while these measures are often effective at ranking different scenarios, it can be extremely difficult to meaningfully explain the practical differences between a ‘good” and a “bad” score. Combined with the arbitrary nature of the threshold this makes it very difficult to explain why a solution is dismissed as ‘bad”, other than a simple “computer says no”. While there is occasionally pushback against this blind reliance on arbitrary thresholds, but it is still a problem that can commonly be observed.

### Privacy-preserving technologies

Approaching privacy as a technical problem has inevitably led to attempts to solve it technologically. In recent years, privacy-preserving or enhancing technologies have been developed to minimize the risk of data leakage and data reidentification. These solutions enable organizations to undertake multi-institution research projects (Scheenstra et al. 2022). They have notably been used to develop various commercial products, such as personalized advertisements, predictive text models for mobile phones, and recommender systems based on users’ profiles and purchase history, but also to improve public services. Hospitals have used Federated Learning to combine patient data in a privacy-preserving manner to train machine learning models for disease diagnosis, which in turn improves healthcare offerings (Kairouz et al. 2021; Li et al. 2020; Yang et al. 2019, 2020; Vatsalan et al. 2017; Truong et al. 2021; Liu et al. 2020; Banabilah et al. 2022).

The progress made in developing these privacy enhancing tools is undeniable. However, current literature is primarily focused on the technical aspects of privacy and ignores other important issues. The focus on technical definitions often means the general public has difficulty understanding what is actually offered to them. Additionally, while mathematical measures of privacy may allow solutions to be ranked easily, this ranking is largely a theoretical exercise, and it may be difficult to determine the exact practical differences between two competing solutions. Lastly, reducing privacy to a purely mathematical problem gives it an “objective” veneer, which can be used to whitewash a project. Additionally, large tech organizations may push their preferred metric in an effort to shape the discussion on privacy in ways that benefit their business model. In the following sections we will elaborate these topics.

## The misplaced focus on preventing data leaks and its consequences

Privacy is important in 4 aspects of the development and implementation of data-driven projects. These aspects are: (1) the training of the model, (2) the use of the model, (3) the technique or technology deployed, (4) the aim and application of the project. It is only when privacy is accounted for in all 4 of these aspects that such a project can be considered “privacy-preserving”. These aspects tend to compete for researchers’ attention. For instance, problems that arise during training (1) might include technical challenges such as data leaks or poisoning attacks by malicious parties. Similarly, issues falling under aspect (2) are primarily technical, such as concerns about model inversion. Additionally, there may be practical problems that may need to be solved regarding the model use, for example where is the model hosted and how it accesses new data. However, the use of the resulting analyses (4) introduces more social and ethical considerations. For example, could the resulting model lead to discrimination, or reinforce existing biases (Verma 2019; Dutch 2021)? Answers to these concerns are often less straight-forward.

Likewise, ensuring the proper technique is applied to a specific problem (3) is relatively straightforward to establish and control. For example, if a project requires zero trust then it is trivial to establish that techniques that rely on a trusted third party are inappropriate. However, determining whether these techniques are implemented in an ethically responsible manner, and will not be abused in the future, for example after a change of leadership, is considerably more difficult. Ethically assessing an algorithm is challenging; but the manner in which it is used deserves attention. As a result, it is common to focus purely on the technical aspects, ignoring the ethical, legal and societal aspects (ELSA), of which privacy is part. Technical data leaks usually result in damage to the data-controller, revealing industry secrets and/or causing organizations to lose their commercial advantage, as well as lead to significant reputational damage and/or fines to the data-controller. This has led to zero-trust policies and complicates large cooperative projects, as sharing data is often deemed too risky or complex to execute safely.

However, these technical leaks do not necessarily lead to real harm for the data subjects. For example, the data leaked might not be directly identifiable without the use of additional information that is only available to the data-controller. While it may be technically possible to combine and cross-reference various external data sources to identify individual data subjects, this is unlikely to be feasible and the risk should be weighed against the effect and probability of successful attack. Additionally, the more sensitive the data, the harder cross-referencing becomes; sensitive data is not only better protected, but also harder to acquire (GDPR 2016).^7^ This greatly limits the real harm done to the data-subjects. Finally, the step from a data leak to personal harm or damages of an individual often requires an active and conscious act of someone, which may not be the case. While it is established that there are multiple dimensions to privacy and we can distinguish between model-controller, model-user and data-subject privacy (Domingo-Ferrer 2007), this distinction is often either ignored or left implicit in technical papers.

To illustrate this, let us look at one of the most famous examples of data reidentification, the Netflix competition of 2006, in which researchers used a freely available public IMDB dataset to re-identify the records contained in the Netflix dataset (Narayanan and Shmatikov 2007). This was despite the fact that the Netflix dataset was considered not to contain any sensitive identifiable data. Additionally, had the IMDB dataset not existed, it would have been possible to create a reference database via phishing attempts, such as using seemingly harmless quizzes on the internet (Parsons 2020), or by abusing data-leaks. In this instance, the risk of reidentification was very high, thanks to publicly available reference datasets, as is clear in hindsight. However, it is important to note that the real harm to data-subjects from this leak was minimal or non-existent as it contained little to no new information compared to the already public IMDB database, and knowing who makes which comments on which movie has very limited risk of damage to that reviewer anyway.

In contrast, a medical dataset does not have an easily accessible public database that could be used to re-identify individuals. It’s also much less likely one would be able to successfully employ phishing websites to gain access to a dataset to cross-reference. No matter how personal the medical data may be, they are, in practice, extremely unlikely to lead to patient reidentification and subsequent harm and damages.

Another example of how minor leaks are often presented as major problems can be seen in the following paper. Slokom et al. claim synthetic data is not privacy preserving because they devised an attack which revealed sensitive data (Slokom et al. 2022). However, they overlook key contextual aspects. Most notably, that the leaked sensitive information is limited and often does not meaningfully improve upon baseline prediction of sensitive attributes; in some scenarios, it even performs worse. Even when successful, the attack only slightly exceeds random performance, achieving about $60\%$ accuracy on a sensitive binary attribute. This high level of uncertainty means that such an attack cannot be deemed a serious privacy threat in this scenario. If attacks with such high levels of uncertainty are deemed major breaches of privacy, publishing any analysis would be impossible, as even basic analyses reveal information (Dwork and Naor 2010; Shokri et al. 2017). While the attack vector is relevant and a potential concern in specific scenarios, Slokom et al. do not address its practical limitations.

These examples are illustrative of the broader problem within privacy-preserving literature caused by the focus on technical research questions and failure to consider contextual practical implications and limitations. These studies usually only consider the worst-case scenario where the attacker has practically unlimited resources, or it is assumed that a reference dataset exists to identify individuals. Each record is presupposed to always be unique enough to be identifiable, even if best practices show that such outliers are uninformative and should be removed from your dataset during preprocessing, thus providing a minimum level of privacy via k-anonymity (Sweeney 2002). Additionally, the impact of an attack is based on the amount of data revealed, not on the contextual harm it can do to the data subject. For example, if an attacker attacks two image recognition models, one trained on faces, one trained on MRI images, and in both cases retrieves an image, and nothing more, this is treated as an equivalent leak with equivalent damage in both scenarios. This ignores the fact that one image may be easier to identify but contains relatively limited sensitive information, while the other image contains a lot of sensitive information but may be more difficult to identify. The potential for harm towards the data subject is vastly different in the two scenarios.

In summary, the real impact on the data subjects in the given context is rarely considered, and neither are their preferences. This leads to a focus on secrecy over privacy. Which in turn also leads to researchers ignoring other important, and often connected, ethical aspects such as the risk of biases harming the data subjects. It also leads to researchers overlooking alternative solutions, such as legal solutions. Lastly, because of this focus on secrecy and the relatively short-term goal of protecting the data-controllers’ interests, researchers and engineers often ignore the long-term implications of a project for the data-subjects.

The focus should instead lie on how the different stakeholders involved are affected, with a strong focus on the data subjects, by potential leaks, as well as how “normal” use of the data would affect them. Additionally, researchers and policy makers should rely less on generic definitions of the risks involved, instead the risks should be estimated on a project-by-project basis. It is important to note that this is not a trivial problem that can easily be automated. Discussing the potential risks and solutions for a particular project is complicated, and will require considerable effort each time. Lastly researchers should acknowledge and actively push for alternative solutions. They should not be allowing privacy preserving technologies to be used to whitewash questionable projects. We will further discuss this practice of whitewashing in the next section.

## The role of big tech in defining what is, should or should not be private

In order to create and maintain a situation where they benefit, Big Tech companies have successfully pushed their own agenda, by influencing our understanding of privacy. This paper has introduced 4 aspects of AI privacy in “The misplaced focus on preventing data leaks and its consequences” section: (1) the training of the model, (2) the use of the model, (3) the technique or technology deployed, and (4) the aim and application of the project. Big Tech companies, however, almost entirely focus their privacy preserving efforts on the first 3 aspects. Indeed, those are the phases that allow them to focus on “objective” technical mathematical problems, for which easily demonstrable solutions can be found. Aspect 4, however, is neglected, as it is more likely to raise questions of a more ethical nature that cannot be addressed straightforwardly. While there are legal frameworks that need to be followed, an application being legal is no guarantee that it is ethical and Big Tech prefers to avoid questions on this topic as much as possible. This attitude is in alignment with the general practices highlighted in “The misplaced focus on preventing data leaks and its consequences” section. This section goes further with that observation, arguing that Big Tech is directly involved in maintaining a reductive understanding of privacy as an issue that can be fixed technologically. This allows them to circumvent deeper questions about their business model. Rather than to rethink the way that they collect and process data, they instead (a) created services advertised as “free” but that users in fact pay for by giving away personal data, (b) hide under the promise of “privacy-preserving” techniques, (c) use these techniques to justify targeted advertising and (d) eliminate dissent and competition through brain draining and lobbying.

### The expectation of “free” online services

Meta, Amazon, Alphabet: all offer services—such as online shopping apps, search engines, and social media apps—that appear free and are so convenient that their use has become the norm. That those services are not ‘free’ for use but paid by the trade-in of personal information is not often clear to users, although awareness has been rising. In the EU, lawmakers have deployed efforts to protect the rights of individuals to their personal data with the General Data Protection Regulation. While the GDPR has, at times, constituted a minor setback or annoyance for these companies, it did not result in them rethinking their incredibly profitable business model. Instead, they opted for privacy-washing and complying in ways that could be qualified as questionable. For example, back in October 2023, Meta announced that it would give its European users the choice to use their platform without being shown relevant ads Facebook and Instagram (2023) and Roush (2023), if they agreed to pay a monthly premium of 9,99€ for the web versions, and 12,99€ for the apps. This was a direct response to the European Data Protection Board (EDPB) warning Meta that they could not force their users to consent to their data being extracted by making them leave the platform if they wished to preserve their data Facebook and Instagram (2023). Yet, the effect was the same: Meta users were greeted with a long wall of text and the choice to tick either one box or the other, deciding whether they wanted to keep using the platform for free, or pay a subscription. This tactic, qualified “pay or okay” Roush (2023), was harshly criticized by the EDPB. It deemed that consent obtained under circumstances where data users are forced to choose between two options—either to allow their data to be used for targeted avertising, or refuse for their data to be processed and thus must pay a fee to keep using the service—without being offered any other alternative cannot be equated to “freely given” consent European Data Protection Board (2024).

### Technological privacy preservation

Aside from offering convenient, attractive and “free” services to their users, Big Tech companies would also suggest that their processing of your personal data is entirely safe and private. While the development of privacy preserving solutions has by no means been limited to just Big Tech, they are heavily involved in the development and promotion of privacy-preserving technologies, in order to sell their point of view. Given that their business model relies on the use of vast amounts of personal data, they have a clear stake in the development of such technologies, as well as their perception by legislative authorities and the public. One of such technologies is federated learning, a term coined by Google, which heavily relied on this technique to develop personalized text prediction in the Gboard, the virtual keyboard with auto-correct and text prediction functionality (Banabilah et al. 2022). Many promotional materials can be found to sing the praises of this learning method, all the while obscuring the fact that the company, although indirectly so, is accessing the contents of our emails, messages, and all other text input processed using Google’s software. Is personalized text prediction worth such an invasion of privacy? The default on our machines would suggest that the anwser is yes.

Given that personal data have become highly commodified and profitable goods, companies naturally try to accumulate as much of it as possible and allow their data scientists to run numerous analyses on it. Hiding behind the promise of privacy-preservation enables this data behaviour: after all, if the data used is anonymous (GDPR 2016),^8^ why should consumers or legislators be worried^9^? However, this anonymity claim is flawed: while privacy preserving techniques (PPTs) can guarantee a certain layer of security for a single analysis, one could run multiple queries concurrently in order to reveal private information. Just because a technology makes data processing safer, does not mean safety is guaranteed. On the contrary, being truly concerned with privacy would mean implementing queries that are predefined and limited in scope for specific high-level functionalities: for instance, building a model. Such an approach would align more closely with EU legislation and its guidelines on ethical and trustworthy AI Proposal for a Regulation of the European (2021), which promote data minimization, human oversight, and prevention of harm. Big tech companies’ tendency to greedily accumulate data directly contradicts these principles.

Rather than fundamentally rethink their unethical business practices, Big Tech has instead focused on advertising technological fixes to the issue of privacy, using objective numbers to solve an issue that is, in fact, societal. As such, the mathematical conception of privacy entirely benefits their agenda. It is much easier to develop new technology to remain under a set privacy threshold rather than to think more deeply about the ethical and safety concerns associated with their processing of personal data. Furthermore, this focus on secrecy and preventing data leaks presents a clear commercial advantage. To ensure that their vision is widely disseminated, Big Tech has funneled a significant amount of energy and funds into research that aligns with their agenda and promotes the use and effectiveness of PPTs (West 2019; Papaevangelou 2024; Zhao et al. 2019; Ochigame 2019). Consequently, views commonly held in privacy-preserving literature tend to align with the interests of these companies and organizations, a point this paper will elaborate on further in Sect. 5.4.

### Privacy preservation and audience targeting

While PPTs afford a higher level of safety in gathering and processing personal data, their use (aspect 4) can result in models which would constitute an intrusion in one’s private life. A blatant example of this is targeted advertising (Srivastava et al. 2023; Antonio et al. 2022; Radesky et al. 2020; Dogruel 2019), which is usually activated by default, and is not easily disabled. Furthermore, allowing your data to be collected is often the condition to access or use most services provided online. Companies claim that they are able to perform such a service while preserving user privacy (Reznichenko and Francis 2014), by using state of the art PPTs. However, it can easily be argued that targeted ads themselves present a breach of privacy. They reflect a user’s browsing history, past purchases, etc. Who hasn’t had the experience of searching something up, only for ads for that very item being plastered all over the next website we visited? Other examples below are empirical evidence of these practices. Expectant mothers are likely to engage with posts and hashtags related to pregnancy, leading to the ads being showed to them being very baby-centric. This could lead to potentially inadvertently revealing pregnancies if one uses their personal device in view of someone else, but it can also lead to personal harm. In 2018, a woman reported that Facebook would continuously show her ads for baby products while she was grieving the loss of her unborn child (Brockell 2018). The company had successfully detected her pregnancy, but not that it had resulted in a stillbirth. With Roe v. Wade being overturned by the Supreme Court in the US in 2022, additional concerns are rising regarding the right to privacy about one’s pregnancy status (Kelly and Habib 2023). The popularity of period-tracking apps is leading to fears that such data might end in the hands of prosecutors trying to enforce the criminalization of abortion. A growing body of literature discusses the many risks associated with Big Tech’s access to information about female reproductive health (Mehrnezhad and Almeida 2023; Shipp and Blasco 2020; De and Imine 2020; Mehrnezhad et al. 2022; Healy 2021). The Cambridge-analytica scandal (Hinds et al. 2020) has also shown that ads can be used to successfully influence democratic elections by targeting the individuals most likely to be swayed (Albright 2016), invading user privacy and using the information collected in the process to manipulate them.

### Biased research and lobbying

A few powerful companies hide behind the label “Big Tech”: arguably the most important are Meta, Alphabet, Microsoft, Amazon and Apple (Verdegem 2024; Affeldt and Kesler 2021). Together, they form an oligarchy, dominating the IT market worldwide. But this influence does not stop there. Recent studies have shown that Big Tech financially backs a large proportion of academics in the field of AI (Project 2023; Abdalla and Abdalla 2021; Menn and Nix 2023; Ochigame 2019). In doing so, they ensure that research aligns with their interest. An example of this is how research is currently being conducted on the topic of “fairly” rewarding data-contributors (Lyu et al. 2020). This would involve rewarding them proportionally to the value of the data they contributed, in a privacy-preserving manner, to a project. Such a system would entirely benefit these oligarchies, as they are the legal custodians of the largest amount of data, and would be completely irrelevant as far as the individual data subjects are concerned as individual subjects will never provide enough data to receive meaningful rewards under such a scheme. It would disproportionately hurt marginalized communities, which already benefit the least from improvements in AI, while suffering the most from its side-effects (Feuerriegel et al. 2020; Arora et al. 2023; Xenophobic 2021). In funding such research, oligopolies gain credibility and build trust by claiming to implement “fair” and “privacy preserving” AI, even though they are clearly serving their own interest, even at the cost of harming others. Consequently, government bodies, who rely on academia for guidance on how to regulate AI, are likely to be influenced by this agenda too Abdalla and Abdalla (2021).

When they are not actively draining brains from academia or from promising startups (Goldman 2024), Big Tech companies actively challenge what little is left of their competition through lobbying. It is interesting to consider the difference in the western public perception of TikTok and Meta, two companies that offer fundamentally similar services with the same modus operandi. That TikTok is facing being banned in the US whereas Meta is allowed to thrive is therefore puzzling, until one becomes aware of the role that the latter played in that situation. It was indeed reported that Meta had spent millions on lobbying for such a ban (Shaw 2024), insisting that the Chinese-owned TikTok represented not only a threat to the privacy of its users, but also to national security, helped in the process by US lawmakers’ long history of sinophobia, which the COVID-19 pandemic only worsened (Siu and Chun 2020). In the EU, the app was banned from the official devices of government personnel for similar reasons (Maheshwari and Holpuch 2024), while the use of US-owned social media platforms remains allowed. This raises the suspicion that Meta rid itself of its biggest competitor on the market, by presenting itself as less of a threat than its Chinese counterpart (Lorenz and Harwell 2022).

## Discussion

The way the issue of privacy is understood, approached and “solved” within the field of machine learning is currently flawed. Rather than serving the interests of the people whose privacy is at risk, arguably it aligns with those of Big Tech companies. They are able to profit from constantly exploiting our personal sphere, extracting as much data as possible, and selling this data to third parties that use this information to either sell us goods and services or, more worryingly, influence our beliefs and behaviour. This situation needs to change. This section goes over potential solutions to some of the problems highlighted above.

First, the understanding of privacy within ML needs to be improved. Too often, invasion of privacy is conflated with data breach. A great deal of effort is deployed in protecting data against such breaches, even when they do not harm data subjects in any tangible way. On the other hand, anonymization techniques are often considered amply sufficient to preserve privacy, despite them not always preventing group or societal harms. As of now, there is a disproportionate focus on data leaks, which is more likely to benefit data-controllers, their trade secrets, and their position of power on the (AI) market, rather than data subjects themselves. Placing further importance on the likelihood of harm and the preservation of data subjects’ control and autonomy is needed to complete the existing vision of privacy preservation in ML. Additionally, acknowledging the fact that privacy itself is a nuanced concept, which cannot entirely be solved through technological means, would be a more honest approach. Acknowledging this limitation would help counter the false sense of security that one’s data can be kept 100% private even when harvested by Big Tech: a false sense of security that these companies will happily use.

Under the current definition of privacy within machine learning, a data leak comprising information about a random unknown patient’s blood type that could very difficultly be linked back to them would be considered a privacy issue, whereas a specific individual woman being labelled as “pregnant” even when she did not voluntarily share that information about herself, might not be considered a privacy issue within the understanding of “privacy” held in machine learning (even though, of course, at law this would be a matter of privacy). This situation is nonsensical and is not beneficial to data subjects.

Secondly, when it comes to European regulation, while steps have been taken in the right direction, they are insufficient to generate change. For example, although consent is often presented as a central mechanism under the GDPR for legitimizing personal data processing, there is much debate around what constitutes freely obtained consent. Indeed, online consent is often obtained through pop-up, sometimes pre-ticked consent boxes to websites and apps that collect personal information (Jablonowska and Michatowicz 2020). Furthermore, as discussed earlier in this article, “pay or okay” subscription models have been developed by companies such as Meta to give users the illusion of agency—although this practice was deemed incompatible with providing a genuine and free choice for users by the EDPB (European Data Protection Board 2024). Still, some controllers may attempt to invoke legitimate interest to do away with consent requirements for scraping personal data for AI model training. Finally, in practice, users often have limited visibility over whether their information was used to train AI models, how to opt out of this process (Grinkevičius 2024; Mauran 2024; Fitzgerald 2024; Hoe Meta Gegevens Gebruikt 2024; Shah 2024), and it is thus difficult for them to become aware of malpractice and enforce their data protection rights. The vision of the EU is short-sighted and fails to address the deeper issues caused by the very business model of these companies. This failure to address the deeper issues, combined with lacking enforcement, has allowed these companies to cleverly avoid making any real change to their unethical practices. Focusing on user consent as a legal basis for data processing does not seem to be the answer when many data subjects have grown accustomed to using free apps and services in exchange for data or feel like they have lost complete control over their personal information. Additional steps in the regulation of AI have been taken, comprising the Digital Market Act, the Digital Services Act and the AI Act, however they are likely to have a similarly limited effect, although perhaps the observed shift (Kusche 2024), in the AI act, to focusing on possible harm and risks to humans, which is one of the 7 guiding principles of trustworthy AI according to EU, could lead to some more promising results. As discussed, data science projects often focus on the training and implementation stages of their models, while neglecting their possible real-life consequences. Becoming more conscious of these long-term implications would make it easier to identify potential risks further down the line. This would not only be an improvement on a project-by-project basis, but also create a healthier culture, where data scientists look beyond their direct responsibilities toward potential future problems. Furthermore, a clearer understanding of the real impact of data leaks onto data subjects, rather than data-controllers, would be beneficial. Current research focusses on theoretical impacts, rather than realistic impact. Identifiable data may be of extremely limited value to attackers, and reidentification itself might not necessarily lead to harm to the data subjects.

Thirdly, while shifting to a risk or harm-based approach rather than a consent-based or technologically private approach would be an improvement, another step in the right direction would be to give more importance to the purpose of data processing. While exceptions to certain GDPR obligations are in place for data processing that is undertaken for research or common good purposes, it is still frequently the cause of much confusion and frustration for many who perform such work. Research has suffered time and time again from attempting to comply with rules for which they were not the primary target. At the same time, EU regulation has failed to significantly hinder unethical and invasive large scale data collection and manipulation by Big Tech companies. Purposes such as targeted advertising and online profiling should be more strictly regulated, especially when they have proven to be harmful. Without a bolder regulatory approach, EU data subjects will continue to see their data being extracted from them and sold to third parties that may not serve their interests. Furthermore, as this is often done without the subjects’ knowledge, existing legal recourse becomes virtually inapplicable: without information about who is accessing or processing one’s data, and for what purpose, how would one request that they cease to do so? Lack of transparency about data handling has led to the inability for people to effectively exercise their rights to control their personal information.

We acknowledge that these are not easy solutions to implement, and will encounter significant opposition, especially from for-profit organizations who do not want to see the status quo change. While it is a bitter pill to swallow, privacy is a complex, context-dependent topic. It is impossible to fully solve within a simple, scalable, generic solution. Accepting the limitations of privacy preserving technologies, and pushing back against those who claim to can solve complex ethical questions with a silver bullet, is the only way forward.

## Conclusion

The machine-learning field has attempted to reduce the complex notion of “privacy” to a purely mathematical, technically solvable problem. This has led to several issues: the creation of privacy thresholds that hold little meaning, the claim that certain technologies will guarantee that personal data will remain private, and an overall focus on the development of such privacy-preserving techniques while completely neglecting longer-term effects of large-scale data-intensive projects. Treating privacy like a simple, solvable issue has allowed the Big Tech oligarchy to continue profiting from harvesting data from millions of users without having to pay sufficient attention to the potential harm caused to data subjects by their activities, justifying their behaviour by advertising their technologically robust privacy-preservation techniques. The steps taken in data protection law so far have not had a significant impact and led to new issues for actors that process data for purposes should instead have been facilitated, such as research. We acknowledge that our critiques clash with the wish for scalable, generic solutions and will invite pushback. Additionally, we acknowledge that our suggested solutions have their own practical limitations. A complex, context-dependent, topic such as privacy simply will not have easy answers.”

It is our hope that this article will spark new discussions surrounding the role of Big Tech, and researchers themselves, in defining privacy not only within the machine-learning field, but also in policy-making and public discourse. These could in turn help reshape the privacy protection framework so that it focuses on those who truly should be protected: the data subjects.

## Footnotes

1 See GDPR, Chapter 8. Specifically, GDPR, Article 83 (4–6), and Article 84.

2 For a contemporary discussion of the range of definitions of privacy, see Roessler, Beate and Judith DeCew, “Privacy”, The Stanford Encyclopedia of Philosophy (Winter 2023 Edition), Edward N. Zalta & Uri Nodelman (eds.) Roessler and DeCew (2023).

3 This is explored in more detail in Townend, D (2024) “Big Data Research: can confidentiality and fiduciary duties fill the gaps in privacy and data protection?” Townend (2025).

4 See Graeme Laurie’s challenging recasting of privacy in Laurie, G. Genetic Privacy: A Challenge to Medico-Legal Norms (Cambridge University Press 2002), Chapter 5 Laurie (2002).

5 Townend, D. (2004) “Overriding Data Subjects’ Rights in the Public Interest.” In Beyleveld, D., Townend, D., Rouillé-Mirza, S., and Wright, J. (Eds) The Data Protection Directive and Medical Research Across Europe. Farnham: Ashgate Publishing Ltd, pp 89–101. Beyleveld et al. (2004).

6 See, for example, Anita Allen, “Genetic Privacy: Emerging Concepts and Values”, in Mark Rothstein (ed.) Genetic Secrets: Protecting Privacy and Confidentiality in the Genetic Era Rothstein (1997).

7 See GDPR, Article 9. Processing of special categories of personal data. See GDPR, recital 51. further clarifying the protection of sensitive personal data, lifting the restriction on processing in cases were explicit consent is provided by the data subject, or: ’[...] for the performance of a task carried out in the public interest or in the exercise of official authority vested in the controller.’. See GDPR, recital 52. which further derogates the processing prohibition of special data for the public interest: ’Such a derogation may be made for health purposes, including public health and the management of health-care services, especially in order to ensure the quality and cost-effectiveness of the procedures used for settling claims for benefits and services in the health insurance system, or for archiving purposes in the public interest, scientific or historical research purposes or statistical purposes’.

8 See GDPR, Article 2(1), respecting Article 4(1), and recital 26. Truly anonymous data, as explained in recital 26, does not fall within the material scope of the GDPR [Art.2(1)].

9 Personal data protection legislation largely does not bite on anonymous—truly de-identified—data; research ethics has always seen consent and anonymisation as the gold standards of protection of the individual. This leaves open other dignity breaches in relation to de-identified data.
